# La Grippe—Its Treatment in Our City

**Published:** 1891-01

**Authors:** 


### LA GRIPPE.

---

*Editors Southern Medical Record:—*

In compliance with your request to differentiate between the degree and character of the symptoms of La Grippe as I observed them this year and last year, I will state that during the prevalence of the disease about one year ago, I noticed a strong tendency to affection of the bronchial mucous membrane and frontal sinuses; in other words resembling closely aggravated common cold. This season I notice a less tendency to involve the bronchial mucous membrane, a greater intensity of febrile symptoms—temperature in nearly all cases above 103 deg. F., profuse perspiration, marked pains in the larger joints, especially of the knees; in early stages constipation, later on diarrhoea: but the most noticeable difference is the great tendency to nausea and vomiting. Last year the disease run its course in from one to three days; this year it lasts from three to ten days. In one case I observed marked inclination to typhoid condition. Last season the disease readily yielded to a saline cathartic and large doses of quinine; this year it does not yield so readily to treatment, but seems to run its course regardless of treatment

Generally, the treatment I have adopted this year is to relieve the pain with an opiate, sulphate of morphine or Dover's powders, and have obtained good results from following:

| | | | |
|---|---|---|---|
| ℞. | Hydrargirum Chl. Mite - - - | | gr. iii. |
| | Podophilin - - - - - | | gr. ss. |
| | Pulv. ipecac. - - - - - | | gr. iss. |
| | Sodii bicarb - - - - - | | gr. xii. |
| M. | Ft. chart No. three. | | |
| Sig. | One every hour. | | |
| ℞. | Qumial sulphatis | | |
| | Acetanilid - - - - - | aa | gr.ix. |
| | Pulv. Capsici. - - - - - | | gr. xxiv. |
| | Camphorae - - - - - - | | gr. vi. |
| M. | Ft. Cap. No. xxiv. | | |

In one or two cases of weakened hearts action, I gave with the above 3-4 gr. of Pulv. Digitalis with each dose.

In most cases I give also the following.

℞. Acidi hydrochl. dil. - - - - gtt. vi.

Elixir lactopeptine - - - q. s. ℥. iv.

M. Sig. Teaspoonful three times a day in half wine glass of water.

I have had no deaths from the disease.

Yours truly,
J. A. CHILDS, M. D.

---

In reference to the treatment of the prevailing influenza, and the many variations, I would say that the beginning of the attack will demand in some respects a different line of treatment, according to the manifestation that is most prominent. My experience has shown that the severest symptoms may manifest themselves either through the nervous system, the upper air passages, or the bronchial mucous membrane. I have found the degree of elevation of the temperature to be very variable, being in some cases slight, while in other the rise may be considerable. Perhaps the most constant of all symptoms is the severer aching, which causes great distress and nervousness. This may be confined to the back, or it may in some cases be so general as to result in a general soreness to the touch of all parts of the body.

Where the suffering is not so great as to require an immediate anodyne, I do not know of anything that gives so good results as a free purgation with calomel and soda, combined with compound extract of colocynth, to be followed if the result is not sufficient, with a full dose of rochelle salts. In every case that I have seen, there has been great sluggishness of the excretions, with great sallowness, and the characteristic yellowness of the sclerotic accompanying what is known as biliousness. In many cases the aching will disappear soon after the free purgation, and there will be great relief from all of the more acute symptoms.

Where there is much involvment of the mucous membranes of the nose and frontal sinuses, I know of nothing that will do more to relieve the condition than the old fashioned "Beards cold powder" representing one grain of opium, four of camphor, and five of carbonate of ammonia. This is not only an excellent sedative to the nervous system, allaying the pain and aching, but by equalizing the circulation, it has a decided effect in relieving the local congestion. In the ordinary colds in the head I have never had it to disappoint me when given in the first congestive stage.

As a general remedy for the purpose of relieving the aching, quieting the nervous system, and producing sleep, without disturbing the secretions, I know of nothing that has given the same result as Phenacetin, given in doses of from five to ten grains, as required. The effect in many cases is almost magical, and as far as I know there have been no unpleasant results from its use. The other "synthetic compounds" such as antipyrin, antifebrine, &c., have a good effect.

Many cases will show the cough as the most prominent symptom, and I know of no treatment other than is employed for the usual bronchial coughs.

One of the most characteristic features of the influenza is the great nervous depression that comes on, sometimes in the beginning of the attack, though in my experience it has been more prominent at the close, when the patient is getting up. A few days of the "grippe" will cause sometimes as much depression as an attack of pneumonia, and this is also accompanied by the most profound depression of spirits, almost to melancholia. In this state I have always gotten a good effect from the use of wine of cocoa, given freely enough to bring back the nervous strength. Of course nutritious diet should be given, and any indication that may present should be treated on general principles.

In spite of any, and all, treatment, the convalescence is in many cases very prolonged and tedious, and in some persons many months fail to bring the usual health.

WM. PERRIN NICOLSON, M. D.

---

The influenza, which is prevalent here now, differs but little from the epidemic of last year, except the attack is accompanied with neuralgia more than before. I rely principally upon Antifebrine and that class of remedies, with small doses of quinine, and I have very little trouble in treating the disease:

℞. Anitfebrine - - - grs. xxiv.
Sulph. quinine - - grs. xvj.
M. Ft. 8 capsules. Sig. One capsule every three hours.

Yours very truly,
N. O. HARRIS, M. D.

---

I have had during the last ten days a number of cases diagnosed by myself as "Grippe." The disease differs from an ordinary cold in several essential features.

1st. Considerable elevation of temperature is usual; this in robust people, is not the rule with an ordinary cold.

2d. General muscular pains with aching, especially in knees and hips is almost universal. Loss of appetite and prostration with slowness of recovery, are out of all proportion to the physical symptoms of the disease.

3d. In not a few cases inflammation of the middle ear has been present, and facial neuralgia has been the rule.

TREATMENT.—Unsatisfactory. Unless the bowels have been recently well moved, I prescribe a cathartic, usually allowing the patient to choose his or her favorite, provided it produces free evacuation. Quinine, I have found of little use—a pill of camphor and opium seems to act well in equalizing the circulation and lessening pain. For neuralgia, when present, I have obtained very good results from both the phenacetine and antipyrine, in 5 grain doses, also using the former to allay fever.

℞. Pulv. opii. - - - grs. v.
Pulv. camphoræ - - grs. xv.
M.—Ft. No. 15. Sig. One every three or four hours.

L. H. JONES, M. D.

---

I do not believe that the cases of the "Grippe," so-called, which are prevailing now, are cases of true influenza. My treatment has been Dover's powder, with calomel and bicarbonate soda. For headaches, phenacetine.

L. B. GRANDY, M. D.

---

In addition to the usual symptoms of pain over the body, fevers and extreme debility, I have notiеed in a majority of the cases of La Grippe, which I have seen recently, a persistent and aggravated gastro-intestinal disturbance—consistent with the idea that it is essentially a catarrhal disease.

The patient suffers alternate attacks of vomiting and diarrhoea, and is hardly able to retain either food or medicine. The fever remits—sometimes almost disappears, when it suddenly returns with renewed violence, bounding up to 103 and 104 degrees F.

In the treatment of La Grippe, I have had the happiest results from a combination of quinine and phenacetine in 3-gr. doses each, every two or three hours. I substitute opium for the phenacetine when diarrhoea supervenes. If there is trouble in retaining, I add capsicum, making a capsule of quinine, 3 grs., powdered opium, 1-3 gr., capsicum, 1 gr.

Expectorants are used if cough is troublesome. The treatment, of course, remains with the character and severity of the symptoms. Stimulants are indicated in many cases.

CLARENCE JOHNSON, M. D.

---

The influenza or La Grippe is paying us another visit. I find that the trouble usually begins with a feeling of being tired, followed in a short time by a severe chill, this followed by a general aching of all the bones in the body.

There is usually a severe cough, accompanied by pain in the chest.

I begin my treatment by giving a good purge at night, followed by quinine in the morning, with applications of cocaine and other local remedies to the nose and throat. The complications must be treated as they occurr. No general rule for treatment can be advised.

FRANK O. STCCKTON, M. D.

---

I regard the present epidemic as old-fashioned influenza or catarrhal fever. I do not consider it La Grippe, as we had it last year. It is in all essentials, milder and more amenable to treatment with less tendency to serious complications and unaccompanied by the great nervous and physical depression characteristic of regular Grippe. All the cases I have so far seen, yield readily to quinine, phenacetine and saline cathartics:

R. Quinia, sulph.,
Phenacetine, aa grs. xxx.
M. Ft. Cups No. xii.
Sig. One capsule every two hours.

WM. C. JARNAGIN, M. D.

---

That La Grippe has re-appeared in Atlanta, is undoubted. In my experience the type is more severe than last year. The febrile movement is more marked and prostration more pronounced. It does not respond to treatment as readily in its management. I use the following:

℞. Sod. Salicyl, ʒ ii55.

Sod. Bicarb, ʒii.

Syr. Zingeber, grs. ℥ii.

Sig. ʒi G. every 4 hours.

Where there are rheumatic pains:

℞. Ammon. muriat, ʒj.

Sin. Ipecac, ʒii.

Morph. sulph, gr. ii.

Syr. Yrln, ℥ s. s.

Aq. camph. q. s., ʒii.

M. ʒi. Give every 4 hours.

CHARRES G. GIDDINGS, M. D.

---

I have seen a number of cases and find the same symptoms present as those which were present in La Grippe last year. There are the same complications. Pleuritis, pneumonitis, etc. I usually prescribe for simple cases:

℞. (1) Phenacetine and Salol, a. a. grs. xl.
M. Ft. Caps No. 11.
Two every 3 hours.
℞. (2) Salicylate zoda, ʒii.
Tr. Digitalis, ʒii.
Syr. Zingeber, ℥j.
Spts. Vini g. a., q. s., ℥iij.
Sig. From 1 to 2 teaspoonfuls every 3 hours.

J. W. DUNCAN, M. D.

We are pleased to see in the city again that clever and pleasant representative of the N. Y. Pharmacal Association, and the Arlington Chemical Co., Mr. E. R. Amis, with his excellent preparations of Lactopeptine, Peptonoids, Phospho-Caffein Comp.

---

We beg to call attention of our readers to our special offer of Medical Books on pages——. Now is the time for you to get your books at reduced rates. We will give any new subscriber the same discount on any book wanted, that is not named in the list.

---

The Columbus Cycle Calendar for 1891, is far ahead of any previous year. It is gotten up in such shape as to make it a useful and ornamental article on the desk of every business and professional man. It is issued by the Pope Manufacturing Co., of Boston, New York & Chicago.

---

The University Medical Magazine, published by the University of Pennsylvania Press, has decided to change its policy somewhat. It has up to the present published nothing but original matter. It proposes now to add a department of abstracts, we might call it, giving the jist of all foreign and home journals. This is to be divided into the various branches with editors for each, as follows : *Medicine*, Profs. Pepper and Tyson; *Surgery*, Profs. Agnew and White.; *Therepeutics*, Prof. Word; *Gynaecology*, Prof. Goodell; *Obstetrics*, Prof. Hirst; and in addition summaries of the various specialties, as, memology, ophthalmology, &c. The size of the magazine is also to be increased, making it one of our most valuable journals.

